# Supplementary material for: Piezoelectricity in hafnia
Source: Nat Commun. 2021 Dec 15;12:7301. doi: 10.1038/s41467-021-27480-5 (PMC8674271; doi:10.1038/s41467-021-27480-5)
Supplement: Supplementary file 1 — Supplementary Information [file 41467_2021_27480_MOESM1_ESM.pdf]

Supplementary Information for: Piezoelectricity in hafnia

Sangita Dutta,[1,2] Pratyush Buragohain,[3] Sebastjan Glinsek,[1] Claudia Richter,[4] Hugo Aramberri,[1] Haidong Lu,[3] Uwe Schroeder,[4] Emmanuel Defay,[1] Alexei Gruverman,[3] and Jorge Íñiguez[1,2]

[1] *Materials Research and Technology Department, Luxembourg Institute of Science and Technology, 5 avenue des Hauts-Fourneaux, L-4362 Esch/Alzette, Luxembourg*

[2] *Department of Physics and Materials Science, University of Luxembourg, 41 Rue du Brill, Belvaux L-4422, Luxembourg*

[3] *Department of Physics and Astronomy, University of Nebraska-Lincoln, Lincoln, NE 68588-0299, USA*

[4] *NaMLab gGmbH, Noethnitzer Strasse 64 a, 01187 Dresden, Germany*

SUPPLEMENTARY NOTE 1: ADDITIONAL EXPERIMENTAL DETAILS, PIEZOELECTRIC CHARACTERIZATION

A. PFM testing of the longitudinal piezoelectric coefficient in La:HfO_2 capacitors

The PFM amplitude signal, A, is proportional to the longitudinal piezoelectric coefficient, $d_{33,\mathrm{eff}}$, as:

$$A = d_{33,\mathrm{eff}} V_0 Q \tag{1}$$

where V_0 is the driving ac voltage amplitude and Q is the quality factor associated with the cantilever dynamics at resonance [1]. The calibration procedure used to evaluate the quality factor and to quantify $d_{33,\mathrm{eff}}$ is described in section C.

The PFM phase signal, on the other hand, is related to the sign of the piezoelectric coefficient. PFM measures the sample contraction or expansion via the converse piezoelectric effect, where the strain developed in the out-of-plane direction, η_3, due to the applied electric field in this direction, E_3, is given by Eq. 2:

$$\eta_3 = d_{33} E_3 \tag{2}$$

$$d_{33} = 2\epsilon_0 \epsilon_r Q_{33} P_s \tag{3}$$

where ϵ_0 and ϵ_r are vacuum permitivity and the relative permitivity of the sample, Q_{33} is the electrostriction coefficient and P_s is the spontaneous polarization [2]. As illustrated In Supplementary Figure 1, in a material with a positive $d_{33,\mathrm{eff}}$, such as PZT [3], the sample will expand (contract) when the applied field and the polarization are in the same (opposite) direction. In PFM, a small ac driving voltage is used to probe the sample expansion and contraction using the lock-in amplifier technique. For a material with positive d_{33}, when the polarization is oriented downward, the sample will expand during the positive half-cycle and contract during the negative half-cycle, thus the sample deformation and the driving ac signal are in phase (top panel in Supplementary Figure 1(a)), while for the upward polarization, there is an 180° phase difference between the driving ac and the sample deformation (bottom panel in Supplementary Figure 1(a)). This scenario was observed in the PZT capacitors (Figure 2(a) in the main text). Following a similar argument, the phase signal will be opposite in a material with a negative $d_{33,\mathrm{eff}}$ –the sample oscillations and the driving field will be in-phase when the polarization is oriented upward and 180° out of phase for the downward polarization (Supplementary Figure 1 (b))–. This scenario is relevant to the PFM phase signal measured in the PVDF film, a well-known negative piezoelectric material [4, 5] (Figure 2(b) in the main text). Under the same measurement conditions, a similar PFM phase signal behavior was observed in the 20-nm-thick La:HfO_2 capacitors (Figure 2(c) in the main text), from which it can be inferred that the sign of the $d_{33,\mathrm{eff}}$ is negative. This behavior was reproducibly observed in multiple capacitors while using different types of cantilevers.

B. Identification of the phase offset and determining the correct PFM phase

During the PFM hysteresis measurement, there could be an uncontrollable instrument-related parasitic phase offset contributing to the measured PFM phase signal, which is typically constant for the same AFM tip and measurement parameters. To identify this phase offset and find out the actual PFM phase related to the piezoelectric deformation of the sample, we have adopted two different approaches: the first one uses a reference sample with a known d_{33} value, while the second approach uses the electrostatic signal from the differential signal between the bias-on and bias-off PFM loops.

In the first approach, a reference sample, a PZT capacitor, is used to obtain the PFM hysteresis loop in the bias-off mode. In general, a measured raw piezoresponse (PR) signal can be represented as

$$\mathrm{PR} = \cos(\varphi - \Delta\varphi) \tag{4}$$

where A and φ are the amplitude and phase signals due to the genuine piezoelectric response, respectively, and $\Delta\varphi$ is a parasitic phase offset. For PZT, which has a positive d_{33} coefficient, the PR signal is in phase with the ac modulation signal for the downward polarization (or out of phase for the upward polarization), so that φ should be equal to zero at the far positive dc bias of the PFM hysteresis loop corresponding to the downward polarization. The parasitic phase offset $\Delta\varphi$ can be found from the actually measured PFM phase signal $\varphi - \Delta\varphi$ and then deduced from all subsequent measurements carried out with the same AFM tip and the same measurement parameters.

In the second approach, PFM hysteresis loops in the bias-on (measured when dc bias is present) and bias-off (measured when dc bias is not present) regimes are measured simultaneously for the sample under investigation. Typically, the AFM cantilever arm is held outside of the top electrode during the measurement, a strong electrostatic interaction between the cantilever arm and the sample will be contributing to the bias-on PFM loop, where the raw piezoresponse bias-on signal ($\mathrm{PR_{on}}$) is a sum of the bias-off PR signal ($\mathrm{PR_{off}}$) and the electrostatic signal, i.e.,

$$\mathrm{PR_{on}} = \mathrm{PR_{off}} + \frac{1}{k}\frac{dC}{dz}V_{\mathrm{dc}}V_0 = A_{\mathrm{on}}\cos(\varphi_{\mathrm{on}} - \Delta\varphi) \tag{5}$$

where k is the AFM cantilever spring constant, dC/dz the cantilever-sample capacitance gradient, V_{dc} the dc voltage, and V_0 the ac amplitude. The term that has a linear dependence on V_{dc} is due to the electrostatic interaction when dc voltage is on, which gives rise to a negative slope versus V_{dc} due to the negative sign of dC/dz. The $\Delta\varphi$ is chosen to satisfy either $\varphi_{\mathrm{on}} - \Delta\varphi = 0$, or $\varphi_{\mathrm{on}} - \Delta\varphi = 180^\circ$ on the far negative V_{dc} side of the hysteresis loop, whichever gives a negative slope for the differential signal ($\mathrm{PR_{on}} - \mathrm{PR_{off}}$), i.e., take the phase offset to be the bias-on phase at the far negative V_{dc} side, and use Eqs. 4 and 5 to calculate the PR_{on} and $\mathrm{PR_{off}}$ signal. If the differential signal ($\mathrm{PR_{on}} - \mathrm{PR_{off}}$) results in a negative slope, this would be the correct phase offset. Otherwise, take $\varphi_{\mathrm{on}} - \Delta\varphi = 180^\circ$ (use the bias-on phase at the far negative V_{dc} side), which would then introduce a negative sign for the ($\mathrm{PR_{on}} - \mathrm{PR_{off}}$) signal. This phase offset $\Delta\varphi$ is then subtracted from the raw PFM phase to obtain the actual PFM phase signal.

Supplementary Figure 2 shows an example of the bias-on (Supplementary Figure 2(a)) and bias-off (Supplementary Figure 2(b)) PFM hysteresis loops obtained on the 20-nm La:HfO_2 capacitors. The raw PR signal is then calculated and shown in Supplementary Figure 2(c), together with the differential signal ($\mathrm{PR_{on}} - \mathrm{PR_{off}}$) that has a linear dependence versus V_{dc} with a negative slope. Removal of a linear slope to the bias-on signal usually leads to the PFM loops very similar to those obtained in the bias-off regime (Supplementary Figure 2(d)).

Both of the two approaches to figure out the correct PFM phase worked well and gave consistent results during our measurements.

C. Calibration procedure for evaluation of the piezoelectric coefficient

The PFM amplitude signal is proportional to the $d_{33,\mathrm{eff}}$ (Eq. 1). An order of magnitude estimate of the $d_{33,\mathrm{eff}}$ for an unknown material can be obtained by comparing the PFM amplitude of the unknown material with that of a reference material [6]. Here, we use the PZT capacitor as a reference sample to calibrate the piezoelectric coefficient of La:HfO_2 in the quasi-static regime, i.e. at a frequency much lower than that used in the dynamic PFM measurements. First, the quasi-static strain loops were measured in the IrO_2/PZT/Pt capacitors by monitoring the deflection signal of the AFM cantilever during application of a triangular voltage sweep at 1 Hz (Supplementary Figure 3(a)). The deflection signal was then converted to the actual displacement of the cantilever through calibration of the optical lever sensitivity using the force-distance curves. From the slope of the quasi-static strain loops, the $d_{33,\mathrm{eff}}$ for the IrO_2/PZT/Pt capacitor was calculated to be 48 pm V^{-1}, which agrees well with values reported in literature [7]. This value is then used to obtain the quality factor of the cantilever using Eq. 1. This allowed us to obtain the calibrated piezoresponse PR loops or the $d_{33,\mathrm{eff}}$-voltage loops for the IrO_2/PZT/Pt capacitors (Supplementary Figure 3(b)). The same cantilever was then used to measure the PFM hysteresis loops in the TiN/La:HfO_2/TiN capacitors. The $d_{33,\mathrm{eff}}$ is calculated from Eq. 1 using the obtained quality factor for the specific AFM tip. The obtained $d_{33,\mathrm{eff}}$ value of $2-5$ pm V^{-1} in the TiN/La:HfO_2/TiN capacitors (Supplementary Figure 3(c)) matches the reported $d_{33,\mathrm{eff}}$ values obtained by means of the interferometric techniques [8–10] and PFM [6].

In addition, the piezoresponse loops will have anti-clockwise rotation in the material with positive $d_{33,\mathrm{eff}}$ (similar to the polarization-voltage loops), while it will exhibit a clockwise rotation in a negative $d_{33,\mathrm{eff}}$. Supplementary Figure 3(c) shows that the PR loop in the TiN/La:HfO_2/TiN capacitors corresponds to a negative $d_{33,\mathrm{eff}}$ coefficient.

SUPPLEMENTARY NOTE 2: LINEAR-RESPONSE FORMALISM

Here we mainly follow the formalism for the response properties of a material introduced in Ref. [11]. Let us consider a reference structure of an insulating crystal with cell volume Ω_0 that is in equilibrium at vanishing macroscopic electric field. We can expand the energy per unit cell volume $E(\mathbf{u}, \boldsymbol{\eta}, \boldsymbol{\mathcal{E}})$ as a function of atomic displacements u_m, homogeneous strain η_j, and applied electric field $\mathcal{E}_\alpha$ around the reference state as follows:

$$E(\mathbf{u}, \boldsymbol{\eta}, \boldsymbol{\mathcal{E}}) = E_0 + A^u_m u_m + A^{\mathcal{E}}_m \mathcal{E}_m + A^\eta_m \eta_m + \frac{1}{2} B^{uu}_{mn} u_m u_n + \frac{1}{2} B^{\mathcal{E}\mathcal{E}}_{mn} \mathcal{E}_m \mathcal{E}_n + \frac{1}{2} B^{\eta\eta}_{mn} \eta_m \eta_n + B^{u\mathcal{E}}_{mn} u_m \mathcal{E}_n + B^{u\eta}_{mn} u_m \eta_n + B^{\mathcal{E}\eta}_{mn} \mathcal{E}_m \eta_n + \text{terms of higher order}\ , \tag{6}$$

where E_0 is the energy density of the reference structure. In this equation the first-order coefficients A^u_m, $A^{\mathcal{E}}_m$ and A^η_m correspond to the forces ($F_m = -\Omega_0 A^u_m$); polarizations ($P_m = -A^{\mathcal{E}}_m$) and stresses ($\sigma_m = A^\eta_m$), respectively. We assume the atomic coordinates and strains are fully relaxed in the reference system. Hence, the coefficients A^u and $A^{\mathcal{E}}$ are zero. The second order coefficients are defined as follows. The force constant matrix:

$$\Phi_{mn} = B^{uu}_{mn} = \Omega_0 \left. \frac{\partial^2 E}{\partial u_m \partial u_n} \right|_{\mathcal{E},\eta} . \tag{7}$$

The purely-electronic elastic tensor:

$$\bar{C}_{mn} = B^{\eta\eta}_{mn} = \left. \frac{\partial^2 E}{\partial \eta_m \partial \eta_n} \right|_{u,\mathcal{E}} , \tag{8}$$

the Born effective charges:

$$Z_{mn} = B^{u\mathcal{E}}_{mn} = -\Omega_0 \left. \frac{\partial^2 E}{\partial u_m \partial \mathcal{E}_n} \right|_{\eta} , \tag{9}$$

the force-response internal strain tensor:

$$\Lambda_{mn} = B^{u\eta}_{mn} = -\Omega_0 \left. \frac{\partial^2 E}{\partial u_m \partial \eta_n} \right|_{\mathcal{E}} , \tag{10}$$

and the purely-electronic piezoelectric tensor:

$$\bar{e}_{mn} = B^{\mathcal{E}\eta}_{mn} = \left. \frac{\partial^2 E}{\partial \mathcal{E}_m \partial \eta_n} \right|_{u} . \tag{11}$$

The barred quantities $\bar{C}_{mn}$ and $\bar{e}_{mn}$ represent the purely-electronic response, and are computed with ionic coordinates to their values at the reference state.

Relaxed-ion tensors

To obtain the physical static response properties one must consider the contribution due to ionic relaxations. We compute such relaxed-ion quantities by introducing the functional

$$\tilde{E}(\boldsymbol{\mathcal{E}}, \boldsymbol{\eta}) = min_{\mathbf{u}} E(\mathbf{u}, \boldsymbol{\mathcal{E}}, \boldsymbol{\eta})\ , \tag{12}$$

obtained from Eq. 6 by setting $\partial E / \partial u_n = 0$. For details on the full derivation see Ref. [11].

Defining

$$C_{jk} = \left. \frac{\partial^2 \tilde{E}}{\partial \eta_j \partial \eta_k} \right|_{\mathcal{E}} \tag{13}$$

and

$$e_{\alpha j} = - \left. \frac{\partial^2 \tilde{E}}{\partial \mathcal{E}_\alpha \partial \eta_j} \right|_{u} , \tag{14}$$

we get the physical elastic and piezoelectric tensors by using Eqs. (7)–(11)

$$C_{jk} = \bar{C}_{jk} - \Omega_0^{-1}\Lambda_{mj}(\Phi^{-1})_{mn}\Lambda_{nk} \ , \tag{15}$$

$$e_{\alpha j} = \bar{e}_{\alpha j} + \Omega_0^{-1} Z_{m\alpha}(\Phi^{-1})_{mn}\Lambda_{nj} \ . \tag{16}$$

In Eq. (16), $e_{\alpha j}$ is also known as the piezoelectric stress coefficient which is defined under the conditions of controlled $\boldsymbol{\mathcal{E}}$ and $\boldsymbol{\eta}$. The piezoelectric strain coefficient ($d_{\alpha j}$) is defined under the conditions of controlled $\boldsymbol{\mathcal{E}}$ and $\boldsymbol{\sigma}$, and is typically what is directly accessible in experiments. $d_{\alpha j}$ is defined as

$$d_{\alpha j} = \left.\frac{\partial P_\alpha}{\partial \sigma_j}\right|_{\mathcal{E}} \tag{17}$$

and is related to $e_{\alpha j}$ as follows:

$$d_{\alpha j} = S_{jk}^{(\mathcal{E})} e_{\alpha k} \ , \tag{18}$$

where S_{jk} ($= C_{jk}^{-1}$) is the compliance tensor.

HfO_2 $a = 5.21$ Å, $b = 5.00$ Å, $c = 5.03$ Å				
Atom	Wyckoff Pos.	x	y	z
Hf	4a	0.466	0.232	0.751
O_I	4a	0.631	0.573	0.888
O_{II}	4a	0.236	−0.041	0.500

Supplementary Table 1. Computed lattice parameters and atomic Wyckoff positions in HfO_2 ($Pca2_1$).

Index	$\bar{C}$	C	$\bar{S}$	S
11	465.3	413.6	2.65	2.99
12	181.3	162.3	−0.78	−0.99
13	151.7	123.4	−0.61	−0.60
22	485.0	407.8	2.59	3.08
23	165.6	132.8	−0.69	−0.73
33	445.7	394.6	2.71	2.97
44	127.0	94.4	7.87	10.59
55	116.7	98.0	8.56	10.20
66	169.3	140.4	5.90	7.12

Supplementary Table 2. Computed elastic (**C**, in GPa) and compliance (**S**, in TPa^{-1}) tensors of the ferroelectric phase of HfO_2. We show the total and frozen-ion (barred) effects. Indices in Voigt notation.

Index	$\bar{C}$	C	$\bar{S}$	S
11	288.3	251.3	4.62	6.93
12	122.5	103.2	−1.41	0.19
13	118.5	74.5	−1.35	−10.24
33	281.7	51.8	4.69	48.72
44	85.7	58.6	11.66	17.05
66	100.2	100.2	9.98	9.98

Supplementary Table 3. Computed purely-electronic $\bar{C}$ (GPa), relaxed-ion C (GPa) elastic tensors and corresponding compliance tensors ($\bar{S}$ and S) (TPa^{-1}) of $PbTiO_3$.

$$\text{Hf} \begin{bmatrix} 5.17 & -0.01 & 0.04 \\ -0.36 & 5.46 & 0.15 \\ -0.04 & 0.23 & 4.99 \end{bmatrix}$$

$$\text{O}_\text{I} \begin{bmatrix} -2.50 & -0.96 & 0.31 \\ -0.76 & -2.94 & -0.68 \\ 0.32 & -0.63 & -2.48 \end{bmatrix}$$

$$\text{O}_\text{II} \begin{bmatrix} -2.68 & -0.15 & 0.25 \\ -0.09 & -2.51 & 0.16 \\ 0.29 & 0.12 & -2.50 \end{bmatrix}$$

Supplementary Table 4. Computed Born effective charges of HfO_2 (in units of electronic charge).

$$\text{Pb}\begin{bmatrix} 3.76 & 0 & 0 \\ 0 & 3.76 & 0 \\ 0 & 0 & 3.46 \end{bmatrix}$$

$$\text{Ti}\begin{bmatrix} 6.20 & 0 & 0 \\ 0 & 6.20 & 0 \\ 0 & 0 & 5.24 \end{bmatrix}$$

$$\text{O}_\text{I}\begin{bmatrix} -2.13 & 0 & 0 \\ 0 & -2.13 & 0 \\ 0 & 0 & -4.43 \end{bmatrix}$$

$$\text{O}_\text{II}\begin{bmatrix} -5.18 & 0 & 0 \\ 0 & -2.64 & 0 \\ 0 & 0 & -2.14 \end{bmatrix}$$

Supplementary Table 5. Computed Born effective charges of $PbTiO_3$ (in units of electronic charge).

$$\text{Pb}\begin{bmatrix} 0 & 0 & 0 & 0 & 3.34 & 0 \\ 0 & 0 & 0 & 3.15 & 0 & 0 \\ 6.89 & 6.89 & 5.01 & 0 & 0 & 0 \end{bmatrix}$$

$$\text{Ti}\begin{bmatrix} 0 & 0 & 0 & 0 & -0.51 & 0 \\ 0 & 0 & 0 & 0.50 & 0 & 0 \\ -3.18 & -3.18 & 30.76 & 0 & 0 & 0 \end{bmatrix}$$

$$\text{O}_\text{I}\begin{bmatrix} 0 & 0 & 0 & 0 & -0.44 & 0 \\ 0 & 0 & 0 & -0.69 & 0 & 0 \\ -5.19 & -5.19 & -34.26 & 0 & 0 & 0 \end{bmatrix}$$

$$\text{O}_\text{II}\begin{bmatrix} 0 & 0 & 0 & 0 & -2.94 & 0 \\ 0 & 0 & 0 & -0.12 & 0 & 0 \\ -2.04 & 3.55 & -0.81 & 0 & 0 & 0 \end{bmatrix}$$

Supplementary Table 6. $\mathbf{\Lambda}$ tensors for the symmetry-inequivalent atoms of the ferroelectric phase of $PbTiO_3$ (in eV Å^{-1}). The 3 rows correspond, respectively, to the 3 spatial directions; the 6 columns correspond, respectively, to the 6 strain indices in Voigt notation.

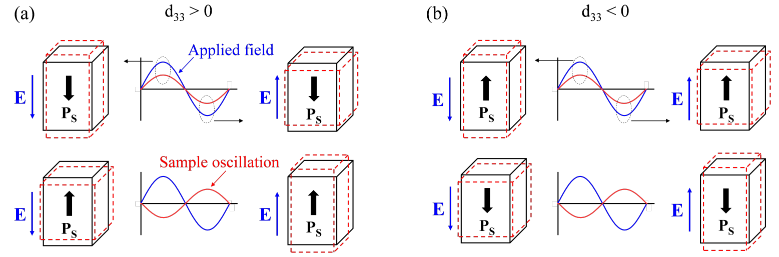

Supplementary Figure 1. Relationship between the applied ac field and the sample oscillation due to the piezoelectric deformation for materials with positive d_{33} (a) and negative d_{33} (b). (a) In a material with positive d_{33}, the sample oscillations and the applied field are in-phase (180º out of phase) when the polarization is pointing downward (upward). (b) In a material with negative d_{33}, the sample oscillations and the applied ac field are in-phase (180º out of phase) when the polarization is pointing upward (downward). The black cuboids indicate the un-deformed state of the domains when no external field is applied, while the red dashed cuboids indicate the deformed state of the domains under an external field due to the converse piezoelectric effect. The blue arrows denote the direction of the applied field, E, and the black arrows denote the direction of the spontaneous polarization, P_s.

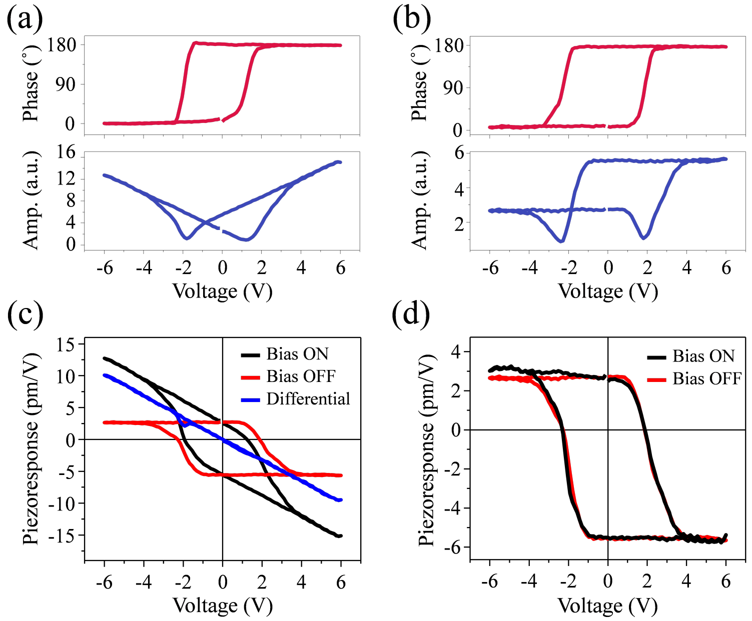

Supplementary Figure 2. Identification of the instrumental phase offset. (a,b) Bias-on (a) and bias-off (b) PFM hysteresis loops in Ti/Pt/TiN/La:HfO_2 (20 nm)/TiN capacitors. (c) Piezoresponse loops constructed from the convolution of the amplitude signal with the cosine of the phase signal for the bias-on (black), bias off (red) and the difference between the bias on and the bias off piezoresponse (blue). The differential signal (blue plot) gives a linear contribution with a negative slope, which can be attributed to electrostatics. (d) Comparison of the bias-on and bias-off PR loops after the linear contribution was subtracted from the bias-on signal.

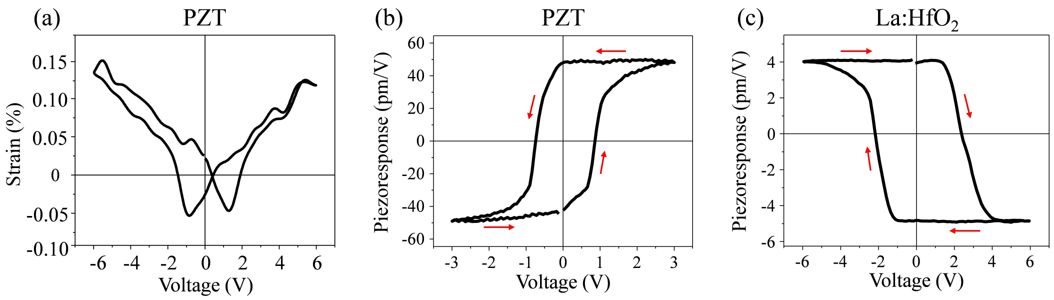

Supplementary Figure 3. Quasi-static strain loop (a) and piezoresponse loop (b) in IrO_2/PZT/Pt capacitors. (c) Piezoresponse loop in TiN/La:HfO_2/TiN capacitors. The red arrows indicate the sense of rotation.

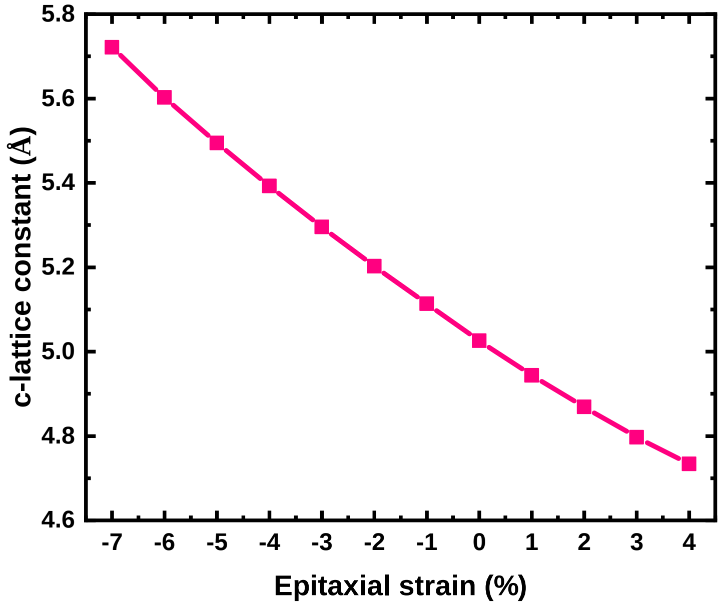

Supplementary Figure 4. Computed c-lattice parameter as a function of epitaxial strain in HfO_2.

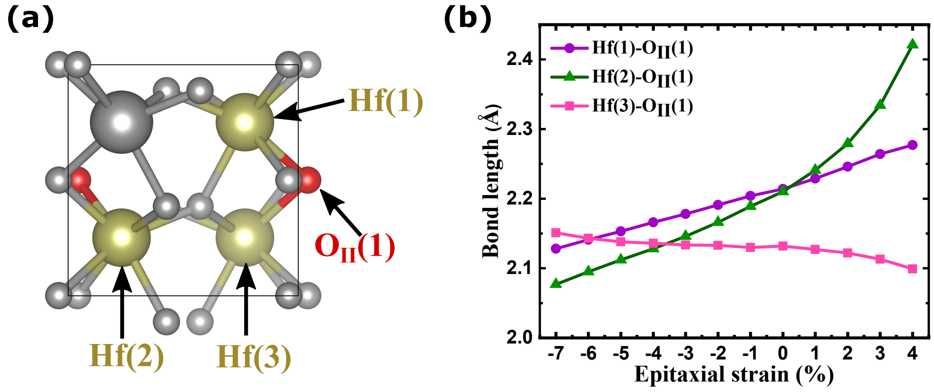

Supplementary Figure 5. (a) Sketch of the FE phase of HfO_2. We highlight one O_{II}-type atom and its three nearest-neighbouring Hf cations. (b) Bond lengths Hf(1)–O_{II}(1), Hf(2)–O_{II}(1), and Hf(3)–O_{II}(1) as a function of epitaxial strain.

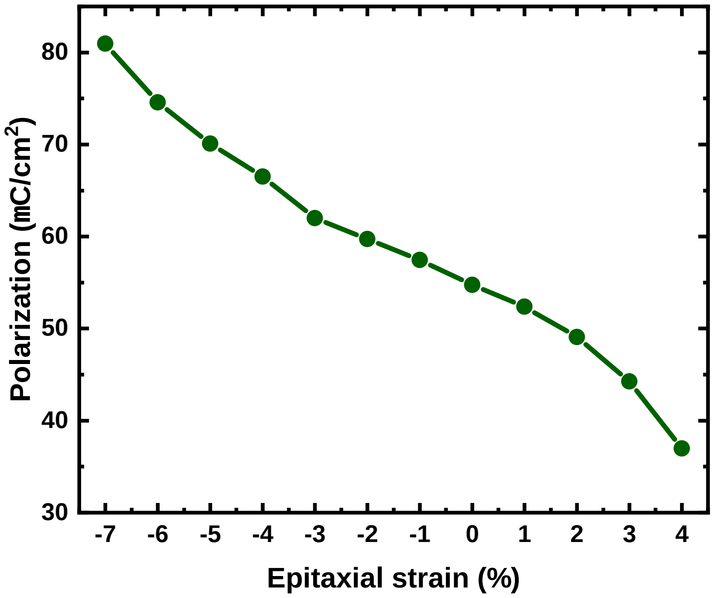

Supplementary Figure 6. Computed polarization as a function of epitaxial strain in HfO_2. The obtained increase of P_3 upon epitaxial compression may seem at odds with the negative value of e_{33}: an epitaxial compression ($\eta_1 = \eta_2 = \eta_{\rm epi} < 0$) leads to an out-of-plane lattice expansion ($\eta_3 > 0$), which yields a positive change of P_3 because $e_{33} < 0$. Note, however, that the total change in P_3 also depends on η_1 and η_2, on account of the (also negative) e_{13} and e_{12} coefficients. Indeed, these transversal piezoelectric effects are responsible for the observed increase of P_3 upon epitaxial compression.

[1] Collins, L., Liu, Y., Ovchinnikova, O. S. & Proksch, R. Quantitative Electromechanical Atomic Force Microscopy. *ACS Nano* **13**, 8055–8066 (2019).

[2] Hong, S. Single frequency vertical piezoresponse force microscopy. *Journal of Applied Physics* **129**, 051101 (2021).

[3] Kholkin, A. L., Akdogan, E. K., Safari, A., Chauvy, P. F. & Setter, N. Characterization of the effective electrostriction coefficients in ferroelectric thin films. *Journal of Applied Physics* **89**, 8066–8073 (2001).

[4] Furukawa, T. & Seo, N. Electrostriction as the origin of piezoelectricity in ferroelectric polymers. *Japanese Journal of Applied Physics* **29**, 675–680 (1990).

[5] Katsouras, I., Asadi, K., Li, M., Van Driel, T. B., Kjær, K. S., Zhao, D., Lenz, T., Gu, Y., Blom, P. W., Damjanovic, D., Nielsen, M. M. & De Leeuw, D. M. The negative piezoelectric effect of the ferroelectric polymer poly(vinylidene fluoride). *Nature Materials* **15**, 78–84 (2016).

[6] Stolichnov, I., Cavalieri, M., Colla, E., Schenk, T., Mittmann, T., Mikolajick, T., Schroeder, U. & Ionescu, A. M. Genuinely Ferroelectric Sub-1-Volt-Switchable Nanodomains in $Hf_xZr_{(1-x)}O_2$ Ultrathin Capacitors. *ACS Applied Materials and Interfaces* **10**, 30514–30521 (2018).

[7] You, L., Zhang, Y., Zhou, S., Chaturvedi, A., Morris, S. A., Liu, F., Chang, L., Ichinose, D., Funakubo, H., Hu, W., Wu, T., Liu, Z., Dong, S. & Wang, J. Origin of giant negative piezoelectricity in a layered van der Waals ferroelectric. *Science Advances* **5**, 1–10 (2019).

[8] Starschich, S., Griesche, D., Schneller, T., Waser, R. & Böttger, U. Chemical solution deposition of ferroelectric yttrium-doped hafnium oxide films on platinum electrodes. *Applied Physics Letters* **104**, 1–5 (2014).

[9] Starschich, S., Schenk, T., Schroeder, U. & Boettger, U. Ferroelectric and piezoelectric properties of $Hf_{1-x}Zr_xO_2$ and pure ZrO_2 films. *Applied Physics Letters* **110**, 2–7 (2017).

[10] Schenk, T., Godard, N., Mahjoub, A., Girod, S., Matavz, A., Bobnar, V., Defay, E. & Glinsek, S. Toward Thick Piezoelectric HfO_2-Based Films. *Physical Status Solidi – Rapid Research Letters* **14** (2020).

[11] Wu, X., Vanderbilt, D. & Hamann, D. R. Systematic treatment of displacements, strains, and electric fields in density-functional perturbation theory. *Physical Review B* **72**, 1–13 (2005).
